# Digital assessment of the accuracy of implant impression techniques in free end saddle partially edentulous patients. A controlled clinical trial

**DOI:** 10.1186/s12903-022-02505-7

**Published:** 2022-11-12

**Authors:** Mohamed M. Dohiem, Medhat Sameh Abdelaziz, Mohamed Farouk Abdalla, Aya Mohamed Fawzy

**Affiliations:** 1 Faculty of Oral and Dental Medicine, Zagazig University, El-Zagazig University, Sharqia, Egypt; 2 Faculty of Oral and Dental Medicine, Prosthodontics Department, Cairo University, Cairo, Egypt; 3 Faculty of Dentistry, Galala University, Suez, Egypt; 4 Faculty of Oral and Dental Medicine, Future University in Egypt, Cairo, Egypt; 5 Faculty of Oral and Dental Medicine, Future University in Egypt, Fifth Settlement, End of 90 street, New Cairo, Cairo Egypt

**Keywords:** Accuracy, Implants, Digital impression, Partially edentulous, Scan body, Custom abutment

## Abstract

**Objectives:**

This in vivo study aims to assess the accuracy of the digital intraoral implant impression technique, the conventional closed-tray impression technique, and open-tray impression techniques in a standardized method of data segmentation along with the best-fit algorithm to overcome the inconsistency of results of previous studies regarding implant impression techniques.

**Materials and methods:**

Sixteen implants were placed in eight patients. Each patient has undergone four impression techniques: direct intraoral scanning of the stock abutment, intraoral scanning using a scan body, conventional closed tray impression technique, and the conventional open tray impression technique. The conventional impressions were poured into stone casts with analogues and stock abutments and scanned using a desktop scanner. In intraoral scanning of the scan body, computer-aided design software was used for the replacement of the scan body with a custom-made abutment that is identical to the stock abutment, allowing comparison with the other impression techniques. The deviation in implant position between the groups was measured using special 3D inspection and metrology software. Statistical comparisons were carried out between the studied groups using a one-way analysis of variance (ANOVA) test.

**Results:**

The total deviation between groups was compared to the reference group represented by the intraoral scanning of the abutment. The total deviation was statistically significantly different (P = 0.000) among the different studied groups. The mean deviation was recorded as 21.45 ± 3.3 μm, 40.04 ± 4.1 μm, and 47.79 ± 4.6 μm for the intraoral scanning of the scan body, the conventional closed, and open tray, respectively.

**Conclusion:**

For implant impressions in partially edentulous patients, intraoral oral scanning using a scan body significantly improves scanning and overall accuracy. Regarding conventional impressions, the closed-tray impression techniques showed more accuracy than conventional open-tray impressions.

**Clinical relevance:**

Intraoral digital implant impression using scan body offers more accuracy than conventional implant impression techniques for recording posterior implant position in free-end saddle partially edentulous patients.

**Supplementary information:**

The online version contains supplementary material available at 10.1186/s12903-022-02505-7.

## Introduction

The most important step in the fabrication of an implant-supported prosthesis with long-term success is to accurately transfer the 3D implant position from the patient’s mouth to the master cast or prosthesis design software [1, 2]. Inaccurate transfer of the implant position results in a poor fit of the prosthesis with biomechanical complications such as bone loss and screw loosening [3, 4]. Many factors affect the implant position on the master cast, such as the impression technique, impression material, tray type, and dental stone manipulation during cast pouring, which are unavoidable human and materials-related factors [3, 5].

With the advancement of computer-aided design and computer-aided manufacturing (CAD-CAM) technology, there are direct and indirect approaches for implant prosthetics digital workflow [6]. The indirect workflow involves making a conventional implant impression which is then digitized by using an optical desktop scanner, while the direct workflow creates a digital scan directly from the patient’s mouth using an intraoral scanner, and scan bodies [6, 7].

Direct digital implant impression has several advantages over conventional impression techniques, including reduced chairside time and increased patient acceptance.[8] It reduces the possible distortion of an impression during impression making and cast pouring [1]. It also eliminates some procedures related to conventional impressions, such as tray selection, pouring of the cast, transportation to the laboratory, and cast storage, as all the data is stored virtually [8–10]. The intraoral scanning procedure is very suitable for patients having problems with conventional impression techniques, such as gag reflex or allergy to the impression material [11–13].

For making a direct digital implant impression, a scan body is screwed onto the implant, then an optical intraoral scanner (IOS) is used to capture the scan body’s 3D position [10, 14, 15]. A specific digital library compatible with each scan body and implant commercial brand is used for virtually determining the exact implant position using superimposition, and a best-fit algorithm [8, 14].

The conventional implant transfers the implant position to the master cast using a rigid tray, an impression coping, and elastomeric material [3]. Several conventional implant impression techniques were proposed for enhancing the accuracy of implant impressions, by splinting impression copings and modifying tray design [3].

Conventional implant impressions can be classified as closed or open-tray impression techniques. The open-tray impression technique utilizes a tray with an open window for unscrewing the impression copings to be removed as one unit with the impression, while the closed-tray impression technique utilizes an impression transfer that remains on the implants while the tray is removed from the mouth. This transfer is later removed from the implant and repositioned into the impression [5, 16]. The closed tray impression technique is indicated in limited inter-arch space situations or where inaccessible posterior implants are present. The conventional impressions could be digitized using desktop optical scanners [5].

To date, different articles have investigated the accuracy of digital implant impression techniques. Tomita et al. [17] reported that the use of an intraoral digital scan for a complete arch impression was more accurate than a conventional impression, in agreement with Arcuri et al. and Alikhasi et al. [18, 19]. On the other hand, Seo and Kim [3] highlighted how the accuracy of digital implant impression decreases in the case of full arch scanning in comparison with conventional impression. However, even if different in vitro studies showed promising results, in vivo studies are currently scarce. etc.

It was reported that clinical data regarding digital and conventional impressions are sparse, and there is a need for valid clinical data on the accuracy of implant impressions as most of the previous studies are in-vitro studies, particularly for partially edentulous arches [2, 4]. Therefore, the primary purpose of this in vivo study was to compare the accuracy between the digital intraoral implant impression technique, the conventional closed-tray impression technique, and the open-tray impression technique. The null hypothesis was that conventional closed-tray and open-tray implant impression techniques have similar accuracy to digital intraoral scans using a scan body for partially edentulous arches.

## Materials and methods

The present study followed the Declaration of Helsinki for the ethical principles of medical research involving human subjects and was approved by the research ethics committee of Future University (FUE-REC (7)/2-2021). The study was registered on clinicaltrials.gov with registration number NCT04908618. first registered on 1/6/2021. Eight male patients with free-end saddle partially edentulous mandibles, with ages ranging from 30 to 50, were selected from the outpatient clinic of the Department of Prosthodontics at Future University Faculty of Oral and Dental Medicine (FUE). The inclusion criteria were patients with missing first and second mandibular molars and a full set of maxillary dentitions with appropriate inter-arch space. All patients have adequate bone width and length for implant placement. All patients were free from any debilitating diseases such as bone diseases, diabetes mellitus, or any other diseases that affect dental implant osteointegration.

### Implant placement

A prosthetically driven implant placement was implemented using a computer-guided flapless surgical protocol. Each patient was scanned using a Cone-Beam Computed Tomography x-ray machine (PaX-i3D Green; VATECH) to obtain a Digital Imaging and Communication in Medicine (DICOM) file. Intraoral scanning of the working arch, the opposing arch, and bite registration was performed using (MEDIT i700; MEDIT Corp) to create a standard tessellation language (STL) file of the patient arches.

After the virtual setting of missing teeth using (Exocad, Dental CAD software), and superimposition of the patient’s DICOM & STL files using (Real guide 5.0 software 3DIEMME), the surgical guides were created.

Two dental implants, 4 mm in diameter and 10 mm in length (Internal hex, conical connection tapered dental implant, IS-II active, Neo Biotech.) were placed in the posterior area of the first and second mandibular molars in each patient according to prosthetically driven implant placement using the surgical guide with copious irrigation. After 6 months, loading of the healing abutments was carried out over the implants for 2 weeks. All the surgical procedures for implant placement were performed by one experienced implantologist.

### Impression techniques

Each patient has undergone four impression techniques, including:

#### Closed tray impression technique

The healing abutments were removed and the closed tray short impression copings were screwed over the implants with a tightening torque of 20 Ncm as recommended by the manufacturer. A bitewing radiograph of the implant is used to eliminate the possibility of a gap between the coping and the implant.

**Fig. 1:**
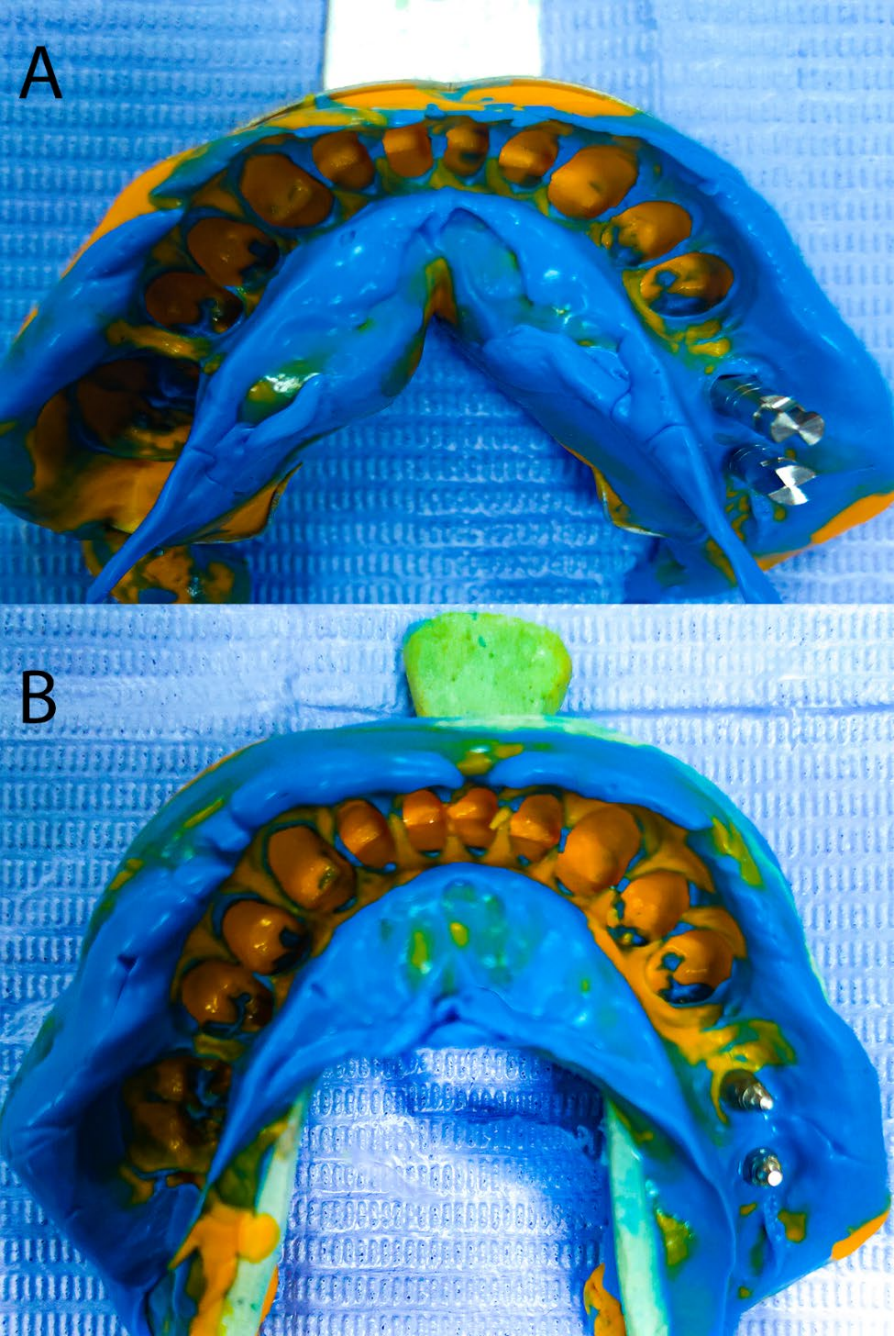
Conventional implant impression techniques. (a) closed tray impression technique. (b) open tray impression technique

A one-step impression technique using hard, stock tray, and putty and light body rubber base (polyvinyl siloxane, Zhermack Elite HD+) was made. The implant analogues were screwed into the impression coping, then the impression coping-analog assembly was positioned into the impression. The impression was poured using a low-expansion type IV dental stone (Zhermack Elite) with an appropriate water/powder ratio according to the manufacturer’s instructions (Fig. 1a).

#### Open-tray impression technique

Open tray impression copings were screwed over the implants with a tightening torque of 20 Ncm as recommended by the manufacturer. A bitewing radiograph of the implant is used to confirm that the impression copings are correctly seated. The impression copings were splinted using dental floss and Dura-lay.

A custom hard tray with open holes over the copings was used to make a one-step impression using Putty and a light body rubber base after applying tray adhesive 10 min before the impression making. After setting the impression material, the impression copings were unscrewed and the tray was removed from the mouth.

The implant analogues were screwed into the impression coping, and the impression was poured into a low-expansion type IV dental stone (Fig. 1b).

To digitize the conventional impressions I and II, the stock abutments were screwed with a tightening torque of 20 Ncm as recommended by the manufacturer on the analogs, followed by digital scanning using an intraoral scanner (MEDIT i700; MEDIT Corp) after being powdered with a homogenous layer of spray (3 M high-resolution scanning spray).

#### Intraoral scanning of stock abutments

Stock abutments identical to those used in conventional impressions were torqued in place intraorally at 20 Ncm, followed by the application of a homogenous layer of optical scanning powder, and scanned with the MEDIT I700 intraoral scanner, producing a digital representation of the abutments directly on the software.

#### Intraoral scanning using scan bodies

Nonmetallic, cylindrical with flattened side scan bodies of 17.1 mm in length (Neo Biotech IS scan body) were screwed onto the implants intraorally with a tightening torque of 20 Ncm, producing a digital representation of the scan bodies and surrounding structures on the software.

**Fig. 2:**
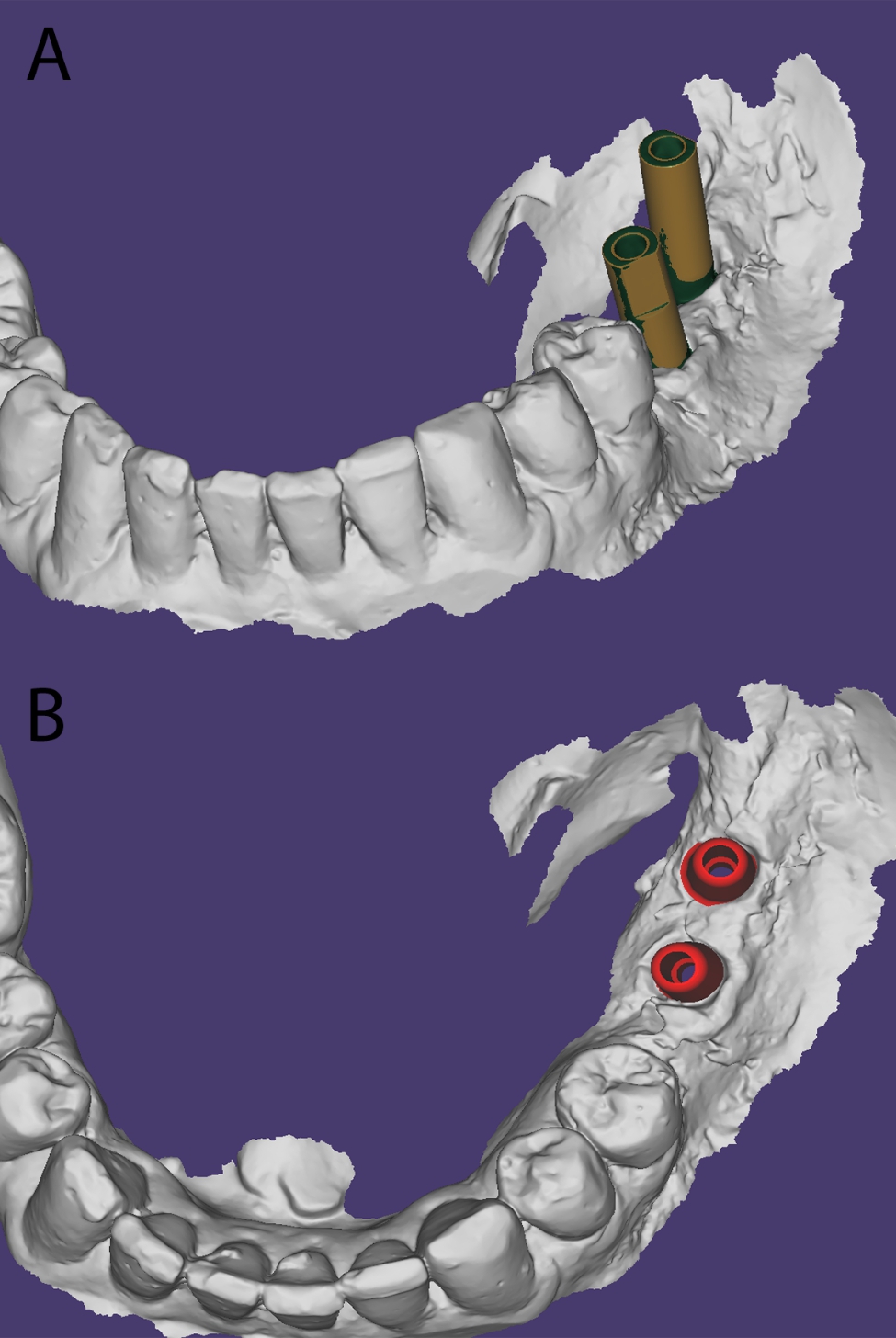
Intraoral scanning of scan body. (a) implant position determination using the superimposition of the scan body from the digital library. (b) digital designing of custom abutments over the implants

A dental computer-aided design (CAD) software (Exocad Dental CAD) was used to fabricate the digital model. Using the best-fit algorithm and a scan body from the digital software library, the exact implant position was determined [20–22], and custom abutments identical to the abutments used in all the other impression techniques were loaded onto the implant (Fig. 2a–b).

For all four studied groups, the scanning protocol was performed in a continuous pattern, starting from the posterior teeth on one side, passing through the anterior teeth, and ending at the stock abutments/scan bodies on the other side. The scan was done over the occlusal surface first, followed by the lingual surface, and ended with the labial and vestibular sides of the teeth. For any missing data, a smooth vertical rotation of the scanner head was made over the defective part of the scanning [23, 24].

The scans were done by one experienced operator in around 5 min for each scan, and then the scans were exported as an STL file.

### The digital comparison of accuracy between implant impression techniques

Using special 3D inspection and metrology software (Geomagic Control X; 3D systems), the intraoral scanning of the stock abutments tightened over the implants was set as a reference model for all upcoming comparisons.

**Fig. 3:**
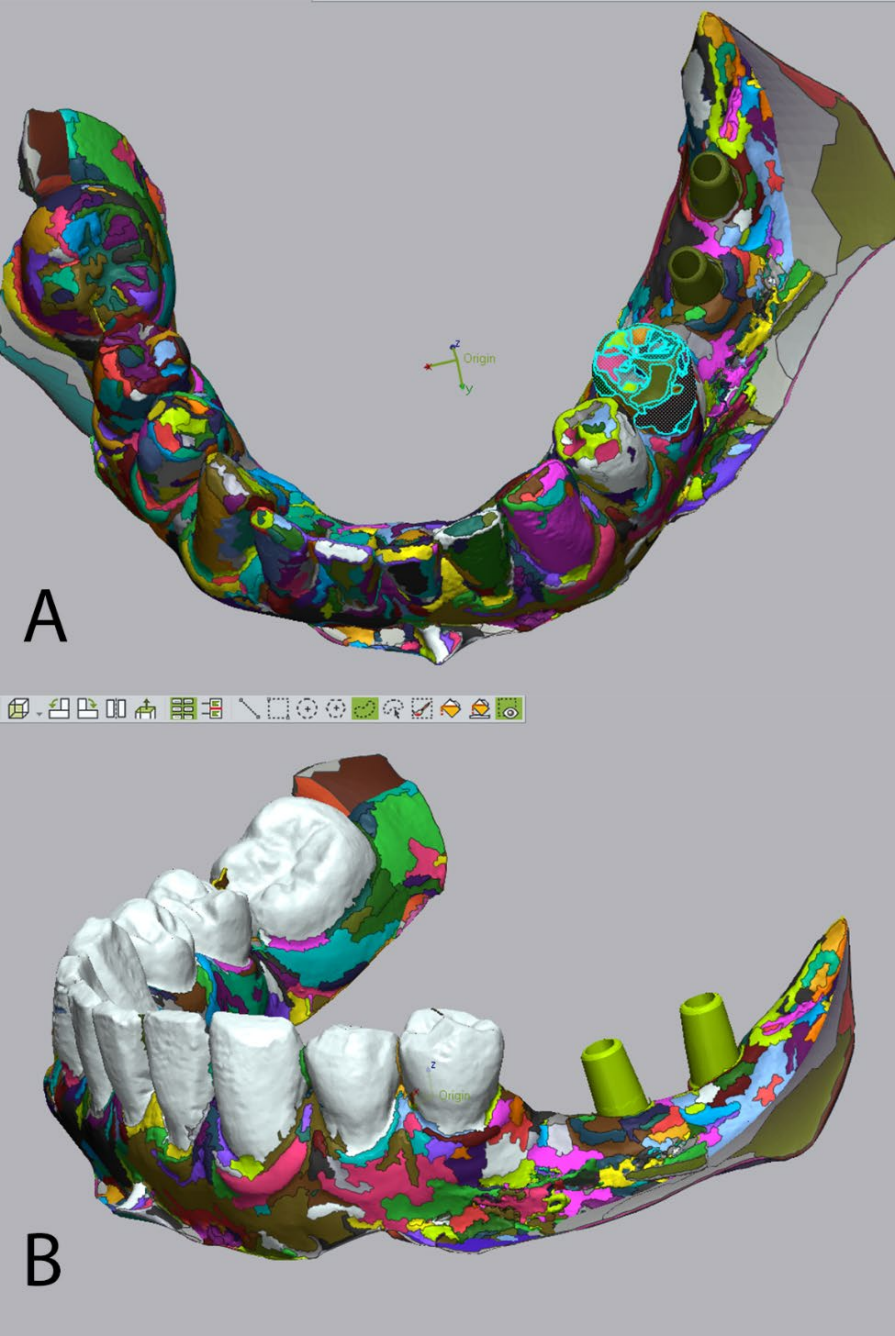
Segmentation of the scanned model into teeth and abutments. (a) the scanned model is composed of multiple segments. (b) segmenting the model into teeth to be used for best-fit superimposition and abutments used for 3D comparison

3D segmentation of the model was carried out to split the model into 2 parts: teeth and abutments. The teeth part is used for superimposition between the models using the best-fit algorithm, while the abutments part is used for 3D comparison of accuracy and deviation in micrometers (Fig. 3a–b).

**Fig. 4:**
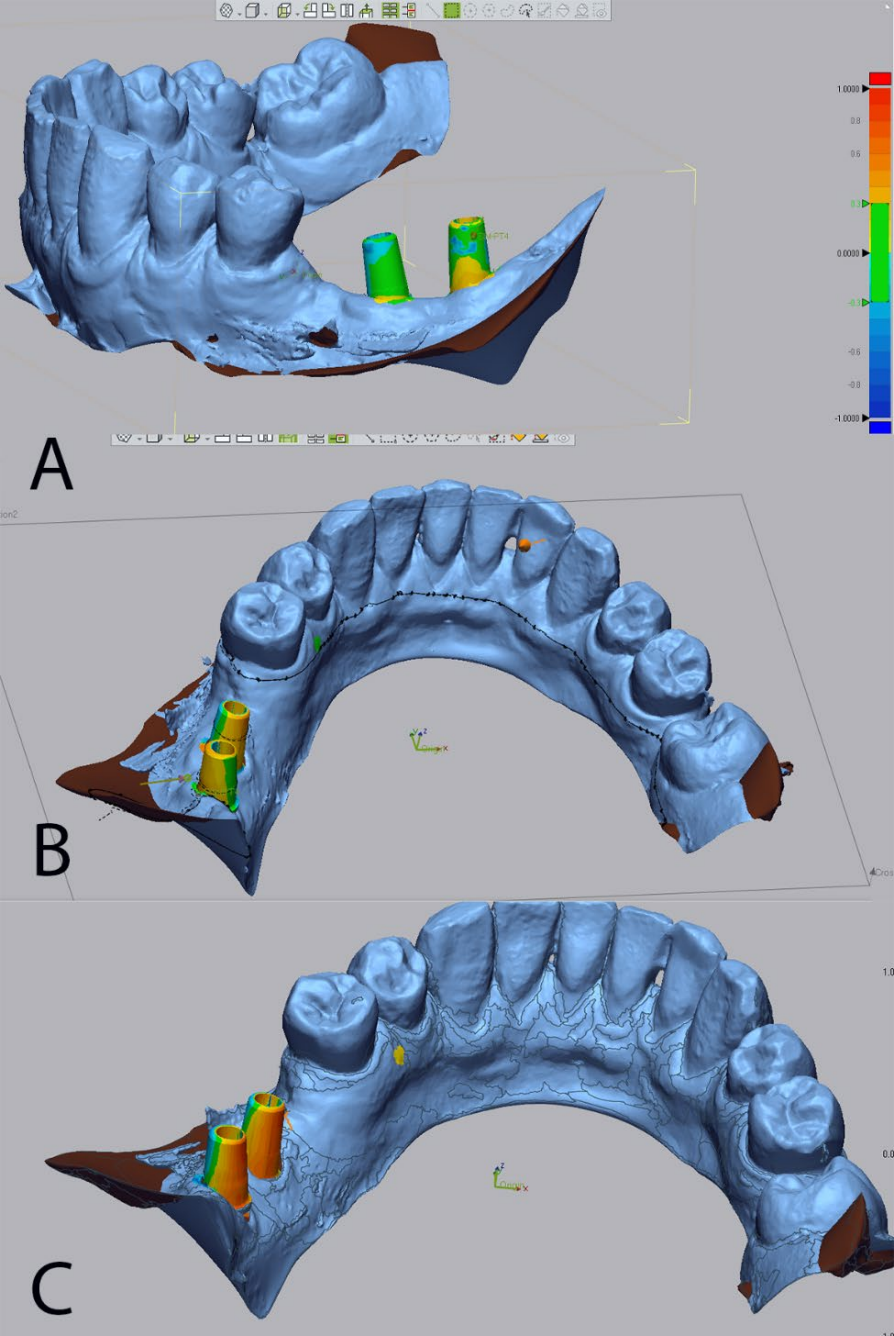
3D deviation of different implant impression techniques. (a) 3D deviation of intraoral scanning with scan body. (b) 3D deviation of closed tray impression technique. (c) 3D deviation of open tray impression technique

The 3D comparison was carried out between the studied groups, and the root mean square (RMS) values representing the total deviation were collected for each group (Fig. 4a–c) (video 1). The RMS value represents the deviation between the data by squaring the distance between the compared data points, divided by the number of data points, which is automatically calculated by the software [10, 25].

**Fig. 5:**
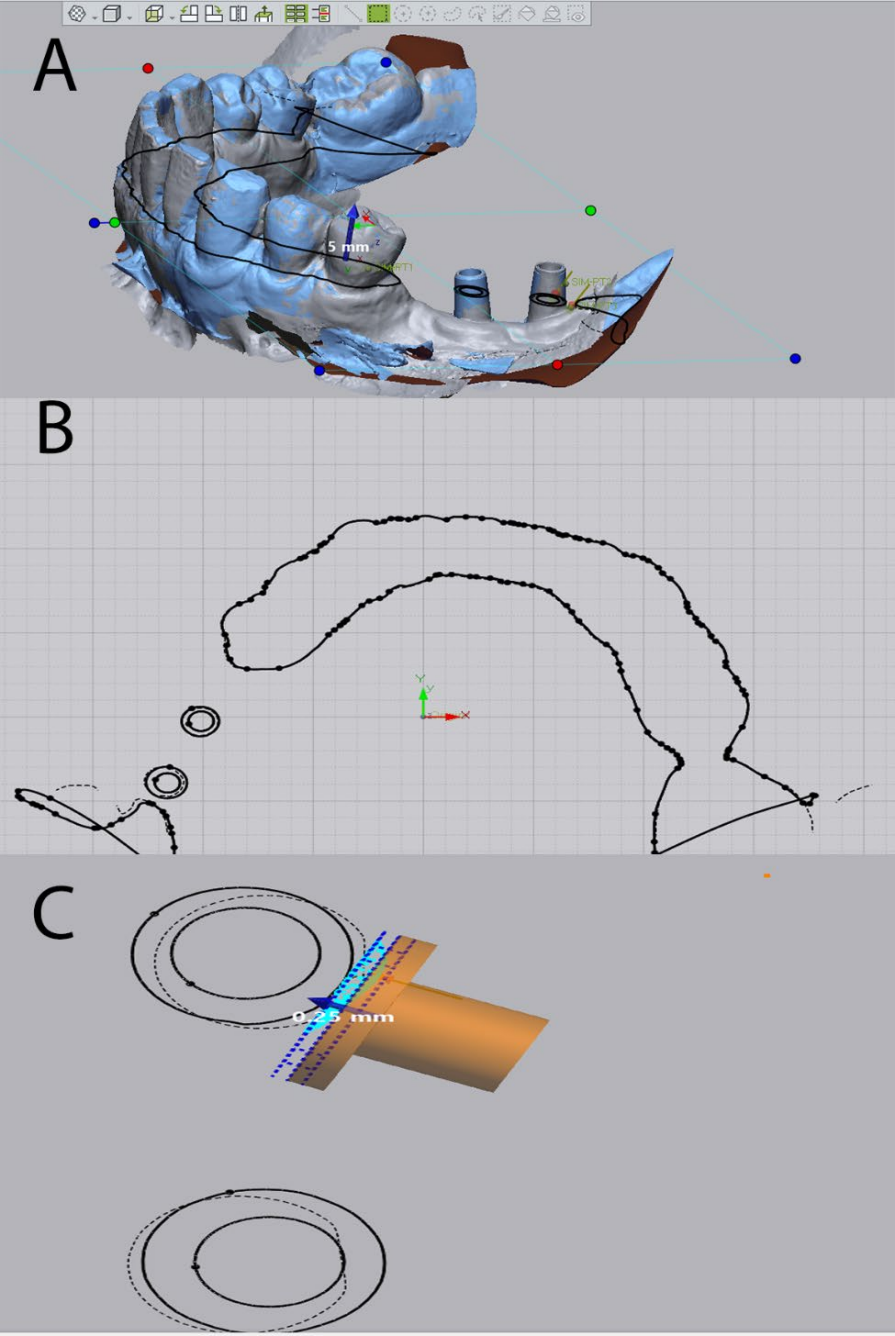
Horizontal split sections in the compared abutments. (a, b) Horizontal split sections are made in all the models at the same Z plane. (c) measuring the distance between the abutments of the superimposed models using the disk contact method

Horizontal split sections are made in all the models at the same Z plane for measuring the distance between the abutments of the superimposed models using the desk contact method (software tool) (Fig. 5a–c) (video 1).

### Statistical methodology

Data were collected and entered into the computer using SPSS (Statistical Package for Social Science) statistical analysis program (ver. 25). Kolmogorov-Smirnov test of normality revealed no significance in the distribution of the variables, so the parametric statistics were adopted. The mean and standard deviation were used to describe the data. Comparisons were carried out between more than two independent, normally distributed subgroups using a one-way Analysis of Variance (ANOVA) test. Post-hoc multiple comparisons were done using the Bonferroni method. An alpha level was set at 5% with a significance level of 95%.

## Results

**Fig. 6:**
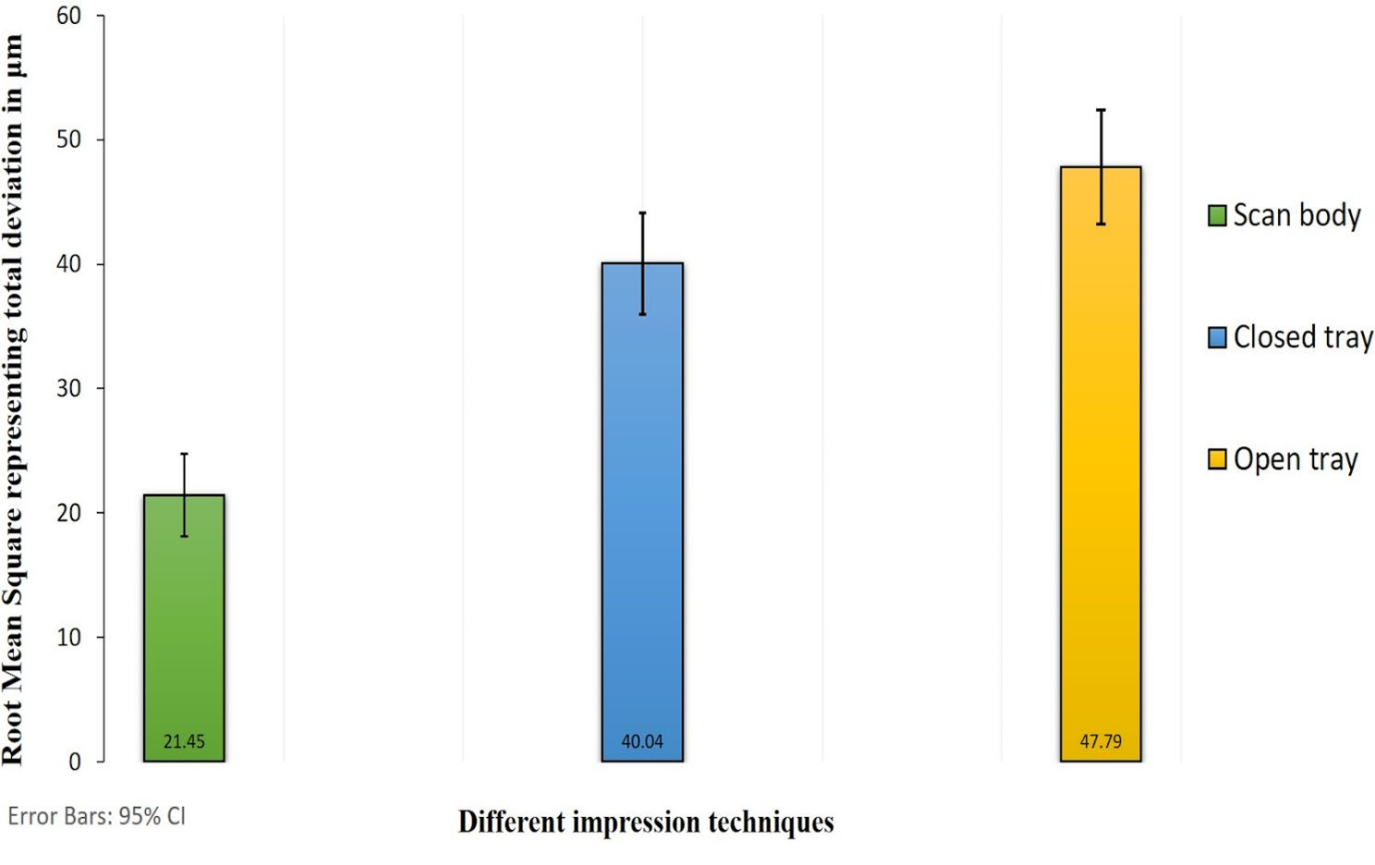
Bar chart comparing the accuracy of the intraoral scanner with scan body Vs. conventional closed and open tray implant impression techniques

The trueness of the different implant impression techniques was shown by the mean and standard deviation of the root mean square values. Intraoral scanning of the scan body and conventional closed and open tray impressions were compared to intraoral scanning of the stock abutment. The mean values of total deviation for the studied groups were 21.45 ± 3.3 μm, 40.04 ± 4.1 μm, and 47.79 ± 4.6 μm respectively (Fig. 6).

The total deviation was statistically significantly different among the three studied groups (p = 0.000). The post-hoc comparison showed a statistically significant deviation between each of the studied groups. Intraoral scanning of the scan body group showed the best accuracy when compared to the control group, while the conventional open tray impression group showed the lowest level of accuracy. Tables 1 and 2.

**Table 1:** One-way ANOVA test comparisons between the three different implant impression techniques

Different impression techniques	Digital impression with intraoral scan body	Conventional closed-tray impression	Conventional open-tray impression	P value
Mean ± SD Total deviation in micro-meter represented by Root Mean Square (RMS)	21.45 ± 3.3	40.04 ± 4.1	47.79 ± 4.6	0.000*
Mean ± SD The distance in micro-meter between the abutments at the horizontal plane	225 ± 0.27.774	236 ± 23.261	267.5 ± 21.213	0.006*

*: Statistically significant (p < 0.05)

NS: Statistically not significant (p>0.05)

**Table 2:** Post hoc multiple comparisons between the three different implant impression techniques

Different impression techniques		P value Total deviation (R.M.S.)	P value Distance between abutments in the horizontal plane
Digital impression with intraoral scan body	Conventional closed-tray impression	0.000*	0.629 NS
Digital impression with intraoral scan body	Conventional open-tray impression	0.000*	0.006*
Conventional closed-tray impression	Conventional open-tray impression	0.003*	0.044*

*: Statistically significant (p < 0.05)

NS: Statistically not significant (p>0.05)

The distances measured between the superimposed abutments at the horizontal cross-section revealed statistically significant (p = 0.006) differences between the groups investigated. The post-hoc comparison showed a statistically insignificant difference between intraoral scanning of the scan body and the closed tray impression technique. (p = 0.629). The conventional open tray impression group showed the lowest level of accuracy at the horizontal cross-section measurements recording (267.5 ± 21.213 μm) (Tables 1 and 2).

## Discussion

Passive fit is a very important factor for the success of any implant-supported prosthesis. For this reason, an accurate impression free from distortion is very critical [8, 26]. In a systematic review, Ting-Shu S and Jian S concluded that digital scans are clinically satisfactory when compared to conventional impression techniques in the fabrication of tooth or implant-supported fixed partial dentures [27]. Introducing intraoral optical scanners (IOSs) into implant prosthodontics has many benefits, such as the elimination of tray selection, impression disinfection, and delivery to the dental laboratory. It also decreases impression distortion during making or on removal from a patient’s mouth, as well as improves patient comfort and acceptance. Moreover, digital scans can be stored electronically as digital information [15, 27].

Kim JE et al. evaluated the accuracy of intraoral scanning of stock abutments and reported that intraoral scanning of stock abutments is an accurate method to fabricate implant-supported prostheses as the stock abutment doesn’t require repeated removal as most impression copings and scan body which could affect the impression accuracy [25]. It was also reported that the intraoral scanners can precisely record the stock abutments’ positional relationship with the elimination of the errors related to different impression materials and techniques [28, 29]. In a study by Natsubori R, it was concluded that Scan powder improves the accuracy in stock abutment scanning, especially in cases of multiple implants [29]. For this reason, the intraoral scanning of implant abutments was set as a reference for all the comparisons in the present study.

IOS scanning of movable tissues such as the tongue and frenum leads to inaccurate results. On the contrary, a better scan is achieved of soft tissue with little or no movement, such as the attached gingiva [11]. For this reason, movable tissues such as the frenum cannot be used as a landmark for superimposition and comparison between different impression techniques; otherwise, false results in evaluating accuracy will occur [11]. The segmentation method used in this study along with the best-fit algorithm guarantee standardization of research results by neglecting any irrelevant data in the comparison, such as soft tissue, which may affect the results of the previous studies.

Accuracy is defined by two factors: trueness and precision. Trueness refers to how close the test scan data is to the reference scan [8, 10, 30]. The best fit alignment method is used to evaluate the accuracy of different digital impressions by scanning either closed or open tray stone cast using a desktop scanner to fabricate a digital model, which was the best fit aligned over the digital models obtained from scanning intraorally using IOS. It was reported that the best-fit alignment method is suitable for the evaluation of accuracy in one quadrant [3, 31, 32]. This algorithm minimizes the global distances between the test and reference data, and the deviation between the data is measured using the root-mean-square error [6]. The further the test data was from the reference, the greater the root mean square and the deviation [30].

In our study, a one-step polyvinyl siloxane impression technique using putty and light rubber base was used for recording closed-tray and open-tray impression techniques. Polyvinyl Siloxane impression material is more accurate compared with polyether impressions in recording implants in a partially edentulous arch [33].

In a study by Ajioka H et al., it was reported that an optical impression has a smaller error concerning the angulation between implants compared to a conventional impression [13]. The results of our study emphasize these findings as the least mean deviations were in the intraoral scanning of the scan body group (21.45 ± 3.3) µm compared with the conventional open tray and closed tray groups.

The findings of the present study showed that the closed tray impression technique was more accurate compared to the open tray impression technique, which can be attributed to its simplicity, and the closed tray impression copings ideal for single and multiple implants impression in patients with inadequate mouth opening for access to the screws retaining the pick- up type impression copings with the impression in place, also in patients with limited inter arch space, the tendency to gag, and implants placed in the inaccessible posterior region of the mouth [34]. The greater accuracy of the closed tray impression technique could also be due to the fact that the path of removal of the impression along with the impression copings in the open tray impression technique which cause deformation of the impression material which will not be fully recovered [14].

The less deviation in the closed tray impression technique coincides with Parameshwari G’s findings, which carried out an in-vitro study comparing different implant impression techniques for partially edentulous patients [33]. It also coincides with Balouch et al., who carried out an in-vitro study comparing the accuracy between the closed tray and open tray impression techniques in 15° angled implants and concluded that the closed tray is more accurate than the open tray technique [35]. Another in-vitro study by Sabouhi M et al. examined the effect of impression techniques and impression coping on the accuracy of impressions and reported that closed-tray impression coping provides a more accurate impression compared to the other coping designs [26].

On the contrary, after an in-vitro study, Izadi A et al. reported that the open tray impression technique provides more accurate impressions compared to other impression techniques [36]. In an in-vitro study comparing the accuracy of different impression techniques in partially edentulous patients by Marghalani A et al., it was reported that there was no difference between open-tray and closed-tray techniques [15]. Concerning the inconsistent results on the accuracy of either the open tray or closed tray method, it appears that the measurement method and superimposition have a great effect on the result [8], there for the segmentation method used in the present study can overcome this problem.

Even though the total deviation between the three implant impression techniques was statistically significant, these deviations were clinically insignificant as they were below the reported deviation for clinically acceptable fit (below 200 μm) [2, 15, 37, 38].

The limitations of this study include in vivo comparison of different implant impression techniques in full arch cases and completely edentulous patients, which can represent a challenge in the intraoral scanning impression technique. The comparison between implant level and abutment level impression techniques is also considered a limitation of this study. This study compares different implant impression techniques in free-end saddle partially edentulous cases. The different implant impression techniques in bonded posterior or anterior partially edentulous patients are also considered a limitation of this study. It is recommended to utilize the adopted methodology in this study in a large sample size, comparing the mandibular and maxillary partially edentulous patients’ different implant impression techniques for different implant placement positions.

## Conclusion

1. Intraoral oral scanning and scan body significantly improve implant impression accuracy.
2. Closed-tray implant impression technique is more accurate than the open-tray impression technique in partially edentulous patients.
3. The introduced method in this research of data segmentation along with the best-fit algorithm has the ability to standardize the measurements regarding implant impression accuracy.

## Electronic supplementary material

Below is the link to the electronic supplementary material.

Supplementary Material 1

Supplementary Material 2

## Data Availability

All data generated or analyzed during the current study are included in this published article and its additional files.

## Declarations

### Clinical trial registration

study was registered on clinicaltrials.gov with registration number NCT04908618.
