# Supplementary material for: Digital assessment of the accuracy of implant impression techniques in free end saddle partially edentulous patients. A controlled clinical trial
Source: BMC Oral Health. 2022 Nov 12;22:486. doi: 10.1186/s12903-022-02505-7 (PMC9655800; doi:10.1186/s12903-022-02505-7)
Supplement: Supplementary file 2 — Supplementary Material 2 [file 12903_2022_2505_MOESM2_ESM.docx]

| comparison | Intra oral scan body | Closed tray scan body | Open tray scan body |
| --- | --- | --- | --- |
| RMS. (total deviation)  In micrometer | 27.73 | 39,56 | 46,46 |
|  | 21,32 | 45,54 | 51,42 |
|  | 22,54 | 33,25 | 42,51 |
|  | 18,45 | 40,02 | 44,51 |
|  | 17,98 | 45,98 | 52,23 |
|  | 24,51 | 38,56 | 54,21 |
|  | 19,64 | 37,54 | 42,31 |
|  | 19,42 | 39,91 | 48,63 |
| Split section measurements. In micrometer | 200 | 250 | 200 |
|  | 220 | 270 | 250 |
|  | 250 | 300 | 260 |
|  | 190 | 240 | 270 |
|  | 230 | 280 | 230 |
|  | 270 | 290 | 220 |
|  | 240 | 260 | 240 |
|  | 200 | 250 | 220 |

**Raw data**
